# Nutritional Supplements Containing *Cardus mariano, Eucalyptus globulus, Gentiana lutea, Urtica urens*, and *Mallotus philippinensis* Extracts Are Effective in Reducing Egg Shedding in Dairy Jennies (*Equus asinus*) Naturally Infected by Cyathostomins

**DOI:** 10.3389/fvets.2020.556270

**Published:** 2020-11-05

**Authors:** Francesca Arfuso, Marilena Bazzano, Emanuele Brianti, Gabriella Gaglio, Annamaria Passantino, Beniamino Tesei, Fulvio Laus

**Affiliations:** ^1^Department of Veterinary Sciences, University of Messina, Messina, Italy; ^2^School of Biosciences and Veterinary Medicine, University of Camerino, Macerata, Italy

**Keywords:** donkey, Cyathostominae, *Equus asinus*, gastrointestinal parasites, phytotherapy, strongyles

## Abstract

The increasing levels of anthelmintic resistance together with the restrictions in the use of drugs in food producing animals have enforced the search for sustainable alternative approaches for parasite control. The current study aimed to investigate the safety and the efficacy of a commercially available phytotherapic formulation against gastrointestinal strongyles in donkeys. Twenty-two Ragusana jennies (2.6 ± 0.5 years old) were assigned to two equal groups. One group was treated with two doses of a phytotherapic supplement Paraxitebio® containing *Cardus mariano, Eucalyptus globulus, Gentiana lutea, Urtica urens*, and *Mallotus philippinensis*, 14 days apart (Group A). One group was used as negative control (Group B). Individual fecal samples were collected at the beginning of the study (T_−1_), and after 7, 14, and 28 days (T_7_, T_14_, T_28_). Blood samples were collected on T_−1_ and T_28_ in order to assess changes in donkeys' hematological profile. After the initial rise in EPG values observed on T_7_, Group A showed a significant EPG decrease with lower eggs per gram (EPG) count compared to Group B on T_28_ and an overall fecal egg count reduction of 56.9% on the same time-point. Hematological parameters were within the normal physiological ranges for enrolled donkeys. However, significant differences in the values of RBCs, Hb, MCHC, MCV, WBCs, eosinophils, and basophils were recorded between groups after phytotherapic treatments, with Group A showing a general improvement in the hemogram picture. The phytotherapic supplement used in the current study was helpful in controlling intestinal parasites allowing a significant reduction in the fecal egg count 28 days after treatment. Further studies are needed to better explore the specific mode of action of the plant-derived formulation herein tested as well as to encourage their use as tool for the control of equine strongylosis under multimodal integrated approach in dairy donkey farms.

## Introduction

In recent years, donkey farming gained popularity in several countries, such as Italy, France, and Belgium, where these equids are mainly reared for milk production (1). Thanks to its special properties, donkey milk is suitable for infants who cannot be breast-fed and people suffering from cow's milk protein allergies (2–4). Donkeys reared for milk production needed to be continuously managed and monitored to maintain optimal general health conditions (5), avoiding nutritional deficiencies (6). Worldwide, gastrointestinal parasite infestations represent a major issue for donkey farming systems. As a matter of facts, helminthiasis is a serious health hazard, inducing poor body condition, poor productive performance, diarrhea, colic, and potentially death in severe cases (7).

Equids are usually coinfected with different nematode species, rather than a single helminth species (8), and cyathostomins, also known as small strongyles, often represent the 95–100% of the total worm burden. These nematodes show a cosmopolitan diffusion, and they are considered as the most important intestinal parasite group in wild and domestic equids for their pathogenic potential at both larval and adult stages (9, 10). The immature cyathostomins can encyst in the large intestinal wall, and it is thought that these stages can persist for years (11). These stages, in particular early third-stage larvae, are relatively insensitive to most anthelmintics available (12). When these larvae reemerge in large numbers from the gut wall, a fatal colitis, named larval cyathostominosis, can occur (13).

Several other nematode species infect equids, although their prevalence is usually lower than cyathostomins (14). Among non-cyathostomin species affecting equids and causing clinical disease included *Parascaris equorum, Strongylus vulgaris, S. edentatus, S. equinus*, and the tapeworm *Anoplocephala perfoliata* [reviewed by (15)]. Also, the pinworm *Oxyuris equi* is relatively common in equids. Donkeys are also susceptible to the fluke, *Fasciola hepatica*, which can be transmitted via snails and the environment, from ruminants. Moreover, the lungworm *Dictyocaulus arnfieldi* is relatively common in donkeys which usually show no disease and can be silent carriers and/or shedders of this parasite, which causes clinical signs in horses [reviewed by (16)].

In the past years, the control of gastrointestinal parasites was based on regular and frequent administration of anthelmintic drugs as preventive treatment strategy. However, the recurring onset of anthelmintic resistance together with the restrictions in the use of drugs in food-producing animals has enforced the search for sustainable alternative approaches for parasite control (17). Among the nutritional supplements used for the control of internal parasites in equine husbandry, promising results have been gained with the employment of plant-derived compounds (18, 19). Although many plants have been listed as having anthelmintic activity in animals (20, 21) and the use of plant-derived anthelmintics would be preferable to synthetic drugs in dairy farming, scientific data demonstrating the real efficacy of these compounds against gastrointestinal parasites are still scarce.

On the basis of the above considerations, the main goal of the current study was to assess the efficacy of 14-day interval administration of a plant-derived product against gastrointestinal nematodes in dairy donkeys. The obtained evidence-based data would improve the current knowledge on the potential use of phytotherapic products for the control of equine strongylosis under the multimodal integrated approach.

## Materials and Methods

### Animals and Study Design

The study was performed in a donkey farm located in Sicily (latitude: 37°23′10″ N; longitude: 14°41′32″ E) in July and August 2019. A total of 22 non-pregnant, non-lactating Ragusana jennies, mean age 2.6 ± 0.5 years, mean body weight 272 ± 27 kg, were enrolled in the study. The study protocol and procedures were approved by the Animal Ethics Committee of Camerino University registration number: E81AC.11/A.

All the donkeys were fed polyphyte meadow hay and had access to pasture 6 h daily, and water was provided *ad libitum*. No anthelmintic treatment had been performed over the 6 months preceding the study.

Fecal samples were collected in the morning (9.00 a.m.), directly from the rectum of each donkey on−1, 7, 14, and 28 days (T_−1_, T_7_, T_14_, T_28_). Samples were transported in a cooled box and analyzed within 8 h from collection. Blood samples (5 mL) were collected by jugular venipuncture into K3-EDTA anticoagulant tubes before phytoterapic administration (T_−1_) and at the end of the experimental period (T_28_). EDTA whole blood samples were delivered to the laboratory and processed within 2 h.

The body weight of each donkey was measured by means of a weighting platform (PS3000HD Heavy Duty Floor Scale, Breckwell, UK).

Enrolled donkeys were firstly grouped into blocks according to their age, body weight, and fecal egg count estimated on T_−1_ and then randomly allocated to two homogenous groups (i.e., Group A and Group B) of 11 animals each.

Animals of Group A were treated with the commercially available phytotherapic product PARAXITEBIO® (BIOEQUIPE SRL, Lombardy, Italy) composed by standardized extracts of *Cardus mariano, Eucalyptus globulus, Gentiana lutea, Urtica urens*, and *Mallotus philippinensis*, and by analytical components including crude protein (0.62%), crude fat (0.22%), crude fiber (0.09%), crude ash (0.32%), moisture (91.32%), and nitrogenous extracts (7.43%).

According to the manufacturer's instructions, the product was administered two times at fortnight interval (i.e., T_−1_, T_14_) using the dose of one syringe (50 g) per donkey.

Donkeys included in Group B were left untreated and served as negative control animals. During the study, the animals were subjected to clinical examinations at each sampling and at the treatment days (T_−1_, T_7_, T_14_, T_28_). The donkeys included in Group A were observed for 12 h after the administration of the phytotherapic supplement in order to record any side effects potentially related with the treatment.

### Hematological Analysis

On blood samples, Red Blood Cells (RBCs), White Blood Cells (WBCs), hemoglobin concentration (Hb), hematocrit (Hct), mean corpuscular volume (MCV), mean corpuscular hemoglobin (MCH), mean corpuscular hemoglobin concentration (MCHC), and platelets (PLTs) were assessed with an automated hematology analyzer (HeCo Vet C, SEAC, Florence, Italy).

For each blood sample, two smears were done and, after air drying the obtained slides, were stained by MGG Quik stain kit (Bio-Optica Milano s.p.a., Milan, Italy). After washing the excess dye from the blood smears and air-drying, the slides were viewed under oil immersion at 100 × by using an optical microscope (Nikon Eclipse Y100; Nikon Instruments Europe BV, Amsterdam, The Netherlands). A manual 100-cell differential count on each blood film was performed by the same laboratory professional. For each animal, the leukocyte differential count was calculated by averaging the data recorded from each blood film of the same sample, and the percentage of lymphocytes, neutrophils, monocytes, basophils, and eosinophils was reported.

### Coprological Examinations and Fecal Egg Count Reduction Test

Fecal egg counts were performed on individual fecal sample by Mini-FLOTAC® technique according to Noel et al. (22) and Went et al. (23). Briefly, five weighed grams of feces were placed into a Fill-FLOTAC homogenizer and suspended in 45 mL of the NaCl flotation medium (specific gravity 1.25). After homogenization, the liquid was transferred into the two 1-mL chambers on Mini-FLOTAC slides. After a 10-min wait time, the top piece of the reading disc was rotated allowing the translocation of the floating eggs to lecture area of the chambers and their counting under 10× magnification (24, 25).

At each time point, two pooled fecal samples per group were incubated at 25°C for 7–10 days for larval development. Third-stage larvae (L3) were recovered using the Baermann–Wetzel technique and identified at species level according to morphological keys proposed by Cernea et al. (26) and by Bowman et al. (27). When a coprocolture had 100 or less L3, all were identified; when a coprocolture had more than 100 L3, only 100 were identified.

Mean values of eggs per gram (EPG) obtained from individual fecal samples on T_−1_, T_14_, and T_28_ were used to estimate the fecal egg count reduction test (FECR) using guidelines established by the World Association for Advancement of Veterinary Parasitology (28) and by the American Association of Equine Practitioners (AAEP) guidelines (29). FERC was calculated according to the formula:

$$\begin{array}{l} \text{FECR}\left(\text{\%}\right)=100\left[\left(\text{C}-\text{T}\right)/\text{C}\right] \end{array}$$

where C is the geometric mean of EPG before the treatment and T is the geometric mean of EPG after the treatment. The geometric mean was calculated by averaging the log counts (x+1) of the single EPG values, taking the anti-logarithm and then subtracting 1.

### Statistical Analysis

All data were expressed as mean values ± standard deviation (±SD).

Data were tested for normality using the Shapiro–Wilk normality test. A normal distribution of the data was found (*P* > 0.05). Two-way repeated-measures analysis of variance (ANOVA) was applied to evaluate the possible significant effects of the phytoterapic treatment and time on the values of EPG and hematological parameters. When significant differences were found, Bonferroni *post-hoc* comparisons were conducted. *P*-values < 0.05 were considered statistically significant. Data were analyzed using statistical software program Prism v. 5.00 (GraphPad Software Ltd., CA, USA).

## Results

None of the animals included in the study showed clinical signs of disease during the experimental period. No adverse reaction nor side effects were observed in animals of Group A following phytotherapic treatments. As shown in Table 1, no differences in body weight values between the two groups were observed at each study time-point.

**Table 1:** Mean values and standard deviation (SD) of egg per gram of feces (EPG) and body weight (BW) recorded in treated (Group A, *n* = 11) and untreated (Group B, *n* = 11) donkeys at study time-points (T_−1_-T_28_).

	**T_−1_**		**T_7_**		**T_14_**		**T_28_**	
	**EPG ± SD (Range)**	**BW ± SD (Range)**	**EPG ± SD (Range)**	**BW ± SD (Range)**	**EPG ± SD (Range)**	**BW ± SD (Range)**	**EPG ± SD (Range)**	**BW ± SD (Range)**
Group A	634.5 ± 280.5 (200–1000)	264.5 ± 29.8 (210–310)	1157.7 ± 429.9 (590–2250)	267.7 ± 28.4 (215–310)	612.7 ± 199.8 (315–950)	270.5 ± 26.8 (220–310)	272.5 ± 84.4 (150–375)	271.4 ± 26.8 (225–315)
Group B	629.1 ± 287.0 (224–985)	280.0 ± 26.1 (250–330)	806.4 ± 475.8 (200–1600)	278.2 ± 26.0 (250–330)	745.0 ± 337.7 (250–1150)	275.0 ± 32.2 (230–320)	835.0 ± 371.3 (350–1400)	274.5 ± 32.7 (230–320)

*Animals in Group A were treated with a phytoterapic product on T_-1_ and T_14_ using the same dose rate*.

EPG values showed no difference between Groups A and B on T_−1_, whereas dynamic changes were observed between the two groups at subsequent time-points and in Group A following plant-derived anthelmintic administration (Table 1).

In Group A, EPG values were higher (*P* < 0.001) on T_7_ than T_−1_ and T_14_, and on T_28_ compared to T_−1_, T_7_, and T_14_. FECR percentages calculated for Group A were −21.6% on T_14_ and 56.9% on T_28_. The EPG values recorded in Group B showed an unchanged trend for most of the study, but not on T_28_ whose values resulted statistically higher (*P* < 0.05) than on T_−1_. Statistical analysis highlighted a positive significant effect of treatment on T_28_ (*P* < 0.001) with Group A showing lower EPG values compared to Group B.

All the L3 harvested at pooled coprocultures carried out on fecal samples of Group A and Group B were identified as Cyathostominae (*Trichonema* spp. and *Poteriostomum* spp.) (Table 2).

**Table 2:** Cyathostominae third-stage larvae developed in pooled fecal samples of treated (Group A, *n* = 11) and untreated (Group B, *n* = 11) donkeys at study time-points (T_−1_-T_28_).

**Cyathostominae species**		**T_**−1**_ (%)**	**T_**7**_ (%)**	**T_**14**_ (%)**	**T_**28**_ (%)**
*Trichonema* spp.	Group A	97.5	99.5	99.4	100
	Group B	96.5	97.5	98.5	97.5
*Poteriostomum* spp.	Group A	2.5	0.5	0.6	0
	Group B	3.5	2.5	1.5	2.5

*Animals in Group A were treated with a phytoterapic product on T_-1_ and T_14_ using the same dose rate*.

As shown in Table 3, hematological parameters evaluated at the beginning and at the end of the study fall within the physiological ranges for donkeys (30). However, significant differences were found for some of these parameters between groups A and B on T_28_. In particular, RBC, Hb, and MCHC values were higher in Group A with respect to Group B on T_28_ (*P* < 0.05), whereas MCV, WBC, and eosinophil values were lower in donkeys from Group A compared to Group B on T_28_ (*P* < 0.001). An increase in RBC values and a decrease in MCV values were found in Group A on T_28_ vs. T_−1_ (*P* < 0.01), while an opposite trend was observed in Group B with lower RBC, Hb, and MCHC values, and higher MCV levels (*P* < 0.05) on T_28_ vs. T_−1_. A significant decrease in WBC, eosinophil, and basophil values together with an increase in lymphocyte number were found on T_28_ vs. T_−1_ in Group A (*P* < 0.001). No time-dependent change was found in the leukocyte population of Group B (*P* > 0.05).

**Table 3:** Mean values ± standard deviation (M ± SD) of red blood cells (RBCs), hemoglobin (Hb), hematocrit (Hct), mean corpuscular volume (MCV), mean corpuscular hemoglobin (MCH), mean corpuscular hemoglobin concentration (MCHC), white blood cells (WBCs) together with the leukocyte sub-population percentages, and platelets (PLTs) determined in treated (Group A, *n* = 11) and untreated (Group B, *n* = 11) donkeys before the first administration of the phytoterapic product (T_−1_), and at the end of the trial (T_28_).

**Parameters**		**T_−1_ M ± SD**	**T_28_ M ± SD**	**Reference range for donkey species^b^**
RBCs ( ×10^6^/μL)	Group A	5.6 ± 1.0	6.4 ± 0.8^*^^a^	4.7–9.0 ( ×10^6^/μL)
	Group B	6.2 ± 1.1	5.3 ± 1.0^a^	
Hb (g/dL)	Group A	10.7 ± 1.2	11.0 ± 1.3^*^	10.6–18.9 (g/dL)
	Group B	10.5 ± 1.7	9.6 ± 0.9^a^	
Hct (%)	Group A	34.5 ± 4.9	34.8 ± 4.9	34–49 (%)
	Group B	35.5 ± 5.3	36.0 ± 5.2	
MCV (fL)	Group A	61.4 ± 2.5	54.3 ± 6.1^*^^a^	46–67 (fl)
	Group B	59.8 ± 5.2	69.4 ± 11.0^a^	
MCH (pg)	Group A	19.4 ± 4.1	17.1 ± 1.3	16–23 (pg)
	Group B	17.2 ± 0.7	18.6 ± 3.1^a^	
MCHC (%)	Group A	32.4 ± 5.4	31.7 ± 2.8^*^	32–36 (%)
	Group B	28.9 ± 2.3	26.8 ± 2.3	
WBCs ( ×10^3^/μL)	Group A	10.8 ± 1.5	8.9 ± 0.9^*^^a^	5.4–15.5 ( ×10^3^/μL)
	Group B	11.2 ± 2.3	12.0 ± 1.5	
Lymphocytes (%)	Group A	40.3 ± 2.8	47.1 ± 6.4^a^	19–67 (%)
	Group B	40.0 ± 6.3	42.8 ± 6.9	
Neutrophils (%)	Group A	42.0 ± 6.2	45.3 ± 5.5	23–69 (%)
	Group B	42.2 ± 5.2	43.8 ± 6.6	
Monocytes (%)	Group A	1.4 ± 1.0	1.6 ± 1.3	0–11 (%)
	Group B	2.2 ± 1.3	2.0 ± 1.7	
Eosinophils (%)	Group A	14.3 ± 6.0	5.5 ± 2.8^*^^a^	0–14 (%)
	Group B	13.9 ± 2.7	10.1 ± 4.0	
Basophils (%)	Group A	2.0 ± 1.4	0.5 ± 0.3^a^	0–1.4 (%)
	Group B	1.7 ± 1.1	1.3 ± 0.8	
PLTs ( ×10^3^/μL)	Group A	266.1 ± 70.5	278.7 ± 55.5	160–584 ( ×10^3^/μL)
	Group B	268.8 ± 64.7	273.3 ± 51.6	

*Animals in Group A were treated with a phytoterapic product on T_-1_ and T_14_ using the same dose rate*.

* *Statistically significant different vs. Group B (P < 0.05)*.

a *Statistically significant different vs. T_-1_ (P < 0.01)*.

b *Weiss and Wardrop (30)*.

## Discussion

The current study provides data on the usefulness of a commercially available phytoterapic supplement to control intestinal strongyle infection in donkeys.

Few published *in vivo* data on the use of phytotherapeutic drugs against gastrointestinal parasites in equids are available in scientific literature, and unsatisfactory results are often found (31). To the best of our knowledge, only one study by Papini et al. (32), investigated the effect of a plant-derived product against gastrointestinal parasites in donkeys but no efficacy has been observed in that report. On the contrary, the phytotherapic supplement used in the current study allowed a 56.9% reduction of intestinal strongyle egg shedding in naturally infected donkeys treated two times at fortnight interval (i.e., T-1, T14).

Recently, the aqueous extracts of *Achillea millefolium* L. (flowers), *Artemisia absinthium* L. (aerial parts), *Centaurium erythraea* Rafn. (flowers), *Gentiana asclepiadea* L. (rhizomes and roots), *Inula helenium* L. (rhizomes and roots), and *Tanacetum vulgare* L. (aerial parts) have been tested *in vitro* for their potential ovicidal and larvicidal activity against donkey nematodes. Except for *C. erythraea*, all tested plant extracts showed significant anthelmintic effects against donkey gastrointestinal nematodes (33). Also, the efficacy of a plant compound containing *Medicago* saponins was tested *in vitro* showing a 90% hatching reduction of donkey gastrointestinal parasite eggs (34). However, the real deworming effectiveness of the *Medicago* saponins has not been assessed in *in vivo* studies, and many factors related to the host and nematode species may alter the bioavailability of the active compound. For instance, host pharmacokinetics may limit the amount of active ingredient reaching the nematodes. The only *in vivo* study carried out on horses infected by strongyles showed that the garlic-derived compound has no effect in reducing the egg shedding (31).

The analysis of pooled coprocoltures indicated small strongyles (Cyathostominae) as the only gastrointestinal parasites infecting the studied donkey population. Specifically, *Trichonema* spp. were found at higher percentage (>96%) compared to *Poteriostomum* spp. (< 4%). No difference in Cyathostominae species was found between treated and control groups on T_−1_ and T_14_. Nevertheless, on T_28_ only *Trichonema* spp. were found in coprocoltures of treated animals. Although only these two Cyathostominae genera have been identified in the studied population, the real presence of gastrointestinal strongyle species may have been suffered for underestimation, since only one-hundred third-stage larvae were identified for each coprocolture. As a consequence, the less spread species belonging to the Cyathostominae subfamily (35–37) may not have been included in the one-hundred larvae identified.

At the beginning of the study, both groups showed very similar EPG mean values, while after two administrations of the plant-derived anthelmintic supplement, a significant reduction in EPG values was observed in Group A (i.e., 272.5 ± 84.4) compared to control Group B (i.e., 835.0 ± 371.3). The results herein gained showed a dynamic EPG trend in treated donkeys. In particular, a significant increase in EPG values was found 7 days after the first treatment compared to the starting pretreatment values, whereas EPG values recorded 14 days after the first product administration showed a decrease with respect to T_7_. Interestingly, a notable EPG reduction was recorded after the second phytoterapic administration estimated on the final time point (T_28_). In the current study, the efficacy of the phytoterapic supplement was evaluated using the method proposed by Nielsen et al. (29) for the synthetic anthelmintic drugs, based on the percentage of egg reduction in fecal samples before and after treatment. The FECR value obtained in the current study is lower compared to other anthelmintic drugs tested in donkeys as ivermectin (96%) (38) and eprinomectin (99%) (39); moreover, according to the AAEP parasite control guidelines, the FECR value herein found is lower than the suggested cutoff values for interpreting results of strongyle FECR in horse (Fenbendazole/Oxibendazole, >95%; Pyrantel, >90%; Ivermectin/Moxidectin, >98%). However, the egg shedding reduction over the fifty percent suggests a potential of the phytoterapic product herein tested as a useful tool for the control of intestinal strongyles in dairy donkeys under the multimodal integrated approach. Furthermore, these findings suggest that the potential anthelmintic efficacy of *Cardus mariano, Eucalyptus globulus, Gentiana lutea, Urtica urens*, and *Mallotus philippinensis* extracts is worthy of investigation also in other equids such as horses, where alternative strategies to chemical products are strongly demanded.

The plant extracts contained in the tested supplement are known to have several anthelmintic properties thanks to their content in terpenoids, steroids, flavonoids, coumarins, and phenols. Particularly, the anthelmintic activity of *Eucalyptus globulus* extracts was investigated *in vivo* in naturally infected sheep showing a FECR of 66% 21 day post treatment (40). *Gentiana lutea* extracts have properties stimulating the immune system and mid-level validity as anthelmintic (41), whereas *Carduus marianus* extracts have choleretic and hepatoprotective actions. Antioxidant, antiviral, and cytotoxic activities have been recognized to the extracts of *Mallotus philippinensis* (42). Indeed, *M. philippinensis* is traditionally used for antifilarial (43), antibacterial, anti-inflammatory, immune regulatory (44), purgative, and anthelmintic (45–47) activities. Scientific reports on the phytochemical analysis of *Urtica urens* have revealed compounds exhibiting anthelmintic, antiviral, immunomodulatory, antioxidant, and anti-inflammatory activities (48–50).

Despite the well-recognized properties of the plant extracts contained in the phytotherapic supplement herein tested, the mode of action of these plant compounds on gastrointestinal nematodes is not well-established. As suggested for other plants constituents like *Allium sativum* extracts, the mode of action could be involved in stimulating the host's immune system in controlling parasites rather than directly killing the nematodes. This hypothesis could explain the rise in EPG values recorded 1 week after the first supplement administration that may reflect an adaptive response of adult nematodes to the changes occurring in their habitat as a result of the potentiated host immune response. The hypothesis that plant-derived supplement used in the current survey can play a role in the immunomodulation of the host seems to be encouraged by the hematological profile shown by treated animals.

Hematological parameters evaluated in the control group and in treated animals before and after phytoterapic product administration fall within the physiological ranges for donkeys. However, at the beginning of the study, in both groups the values of Hb, Hct, MCH, and MCHC were close to the lower limit of the reference range, while the values of MCV, WBCs, eosinophils, and basophils were close to the upper limit of the reference range established for donkey species (30). As previously observed in a mule infected by Cyathostominae (51), also the hemogram picture obtained in this study could be related to parasitism. In fact, the high values of eosinophils and basophils could indicate an active inflammatory response to parasitic invasion and larval migration (51–53). Noteworthily, after the second administration of the phytotherapic supplement, treated donkeys exhibited an improvement of the general hemogram picture on T_28_ with a slight increase in RBC and Hb values and a marked decrease in eosinophil and basophil number, suggesting a moderate attenuation of inflammatory response.

According to the above findings, it can be speculated that the phytotherapic supplement used in the current study has an immunomodulatory effect on host's immune response either by attenuating inflammation or by increasing the host's ability to cope with parasitic growth and proliferation.

## Conclusions

Nowadays, the recurring onset of resistance to existing drugs by intestinal strongyles of equids and the increased public awareness for drug residues in animal products compel the scientific community to investigate novel strategies to control parasitic diseases in domestic animals.

According to the findings herein obtained, the use of phytotherapic supplement could represent a useful tool in the integrated/biological control of parasites in dairy donkeys. Two administrations of phytotherapic supplement at fortnight interval were successful in reducing 56.9% intestinal strongyle egg shedding in naturally infected donkeys, causing no adverse reaction in treated animals throughout the experimental period. Further studies are, however, needed to better explain the mode of action of plant-derived products and to suggest their proper employment as tool for the control of equine strongylosis under the multimodal integrated approach.

## Data Availability Statement

All datasets presented in this study are included in the article/supplementary material.

## Ethics Statement

The study protocol and procedures were approved by the Animal Ethics Committee of Camerino University registration number: E81AC.11/A. Written informed consent was obtained from the owners for the participation of their animals in this study.

## Author Contributions

FA, MB, and EB conceived and designed the study. MB performed the veterinary examinations and sampling. FA and GG carried out the laboratory work. FA drafted the first version of the manuscript. MB, EB, GG, BT, AP, and FL critically reviewed the manuscript. All the authors read and approved the final manuscript.

## Conflict of Interest

The authors declare that the research was conducted in the absence of any commercial or financial relationships that could be construed as a potential conflict of interest. The reviewer VV declared a past co-authorship with some of the authors MB, BT, and FL to the handling editor.

## Abbreviations

RBCs: Red Blood Cells
WBCs: White Blood Cells
Hb: Hemoglobin concentration
Hct: Haematocrit
MCV: Mean Corpuscular Volume
MCH: Mean Corpuscular Hemoglobin
MCHC: Mean Corpuscular Hemoglobin Concentration
PLTs: Platelets
EPG: eggs per gram
FECR: fecal egg count reduction test.
